# Green preparation of hydrogel particles‐in‐emulsions for simultaneous enhancement of humoral and cell‐mediated immunity

**DOI:** 10.1002/elsc.202000011

**Published:** 2020-09-21

**Authors:** Yongjuan Zou, Shuai Li, To Ngai, Songping Zhang, Guanghui Ma, Jie Wu

**Affiliations:** ^1^ State Key Laboratory of Biochemical Engineering, Institute of Process Engineering Chinese Academy of Sciences Beijing P. R. China; ^2^ School of Chemical Engineering University of Chinese Academy of Sciences Beijing P. R. China; ^3^ Department of Chemistry The Chinese University of Hong Kong Shatin NT Hong Kong; ^4^ PLA Key Laboratory of Biopharmaceutical Production and Formulation Engineering Institute of Process Engineering, Chinese Academy of Sciences Beijing P. R. China; ^5^ Jiangsu National Synergetic Innovation Center for Advanced Materials Nanjing P. R. China

**Keywords:** cellular immune responses, green preparation, humoral immunity, oil‐in‐water emulsions, quaternized chitosan particles

## Abstract

Emulsions are one of the most often used vaccine adjuvant formulations. Although they promote high humoral immunity, the induced cellular immunity is often poor, which restrict their application. To enhance the cellular immunity, some researchers have prepared mixed formulations by adding particles into the aqueous phase of emulsions. However, the particle preparation process usually involves the addition and removal of organic reagents, which is environmentally unfriendly and cumbersome. Moreover, the obtained vaccine adjuvant only induces limited cell‐mediated immunity and humoral immunity compared with emulsion‐adjuvanted vaccines. Herein, we developed a green and simple method for fabricating a novel nanoparticles‐in‐emulsions (NPE) formulation. Firstly, a temperature‐sensitive hydrogel was used to prepare particles by self‐solidification without additional crosslinking reagents. Secondly, the white oil was used as organic phase to avoid the particle washing procedures and organic solvent residues. Moreover, the effect of NPE as vaccine adjuvant was evaluated by using two veterinary vaccines as model antigens. NPE showed advantages than the conventional vaccine formulations in inducing both humoral and cellular immunity. This work provides a facile and broadly applicable approach for preparing nanoparticles‐in‐emulsions formulation, and presents an effective adjuvant for enhancing immunity against infectious diseases.

**Abbreviations**
BMDCs: bone marrow‐derived dendritic cells
FMD: foot‐and‐mouth diseases
GP: α, β‐glycerohosphate
NP: nanoparticles
NPE: nanoparticles‐in‐emulsion
PDI: polydispersity index
PRRS: porcine reproductive and respiratory syndrome
VLPs: virus‐like particles
W/O/W: water‐in‐oil‐in‐water

## 1 INTRODUCTION

Animal infectious diseases, especially zoonotic infectious diseases, pose serious threats to the development of livestock husbandry and health of occupational personnel [1]. Throughout history, zoonoses such as bubonic plague, rabies, and influenza have killed millions of people, resulting in devastating disasters to humanity [2]. These events remind us of the importance of developing safe and effective vaccines for preventing the transmission of infection [3]. Moreover, since adjuvants can significantly increase the magnitude and duration, or alter the types of immunity, they have become indispensable components in vaccines [4].

The adjuvants commonly used in veterinary vaccines are white oil‐based emulsions, especially water‐in‐oil‐in‐water (W/O/W) emulsions [5]. Although W/O/W emulsion adjuvants can evoke high humoral immunity, they cannot induce effective cellular immune responses [6], which were found to be important for preventing potential infections and clearing mutant viruses [7]. Currently, there are various particles under development that can promote excellent cellular immunity [8]. Thus, to improve both humoral and cellular immune responses, complex formulations created by combining particles with emulsions have been reported in a few papers [9, 10]. However, in order to prepare such formulations, particles and emulsions need to be prepared separately before mixing. Moreover, particles are usually produced by emulsification techniques that use organic solvents as the oil phase, and contains a tedious washing process [11]. In this process, harmful solvent residues and high particle loss during washing are also inevitable. Furthermore, simply mixing particle and emulsion adjuvants, which are effective independently in vaccine formulations, does not result in enhancement of both humoral and cellular immunity.

Herein, we present a green preparation method for generating a particles‐in‐emulsions adjuvant. Specifically, we utilized a quaternized chitosan‐based solution, which can thermally self‐solidify to form hydrogel particles without adding additional crosslinker [12]. The premix membrane emulsification technology was used to produce particles of a specific size. Moreover, by directly adopting white oil, an adjuvant often used for veterinary vaccines, as the oil phase, the particle preparation and emulsion preparation processes could be seamlessly connected. The entire process was completed without additional cumbersome particle washing steps and without using unnecessary additives, particularly organic solvents. In addition, we proposed this new adjuvant, a particles‐in‐oil‐in‐water emulsion (NPE), to explore the synergistic immune effects with two kinds of typical antigens. One was the inactivated antigen for porcine reproductive and respiratory syndrome (PRRS), and another was the virus‐like particle (VLP) antigen for foot‐and‐mouth disease (FMD). By examining serological antibodies and lymphocyte subpopulations in spleen cells, the adjuvanticity of NPE was further compared to that of ISA 206.

## 2 MATERIALS AND METHODS

### 2.1 Materials and equipment

Quaternized chitosan (Degree of quaternization DQ ≈ 40%) was synthesized according to our previous study [13]. α, β‐Glycerohosphate (GP) was bought from Kaiyuan pharmaceutical & Chemical (Shanxi, China). White oil was provided by Petro‐Canada Lubricants Inc. (Ontario, Canada). PO500 and Tween80 were from Sakamoto Yakuhin Kogyo (Osaka, Japan) and Merck & Co. Inc. (Darmstadt, Germany), respectively. Membrane emulsification equipment and porous membranes were obtained from Zhongke Senhui Microsphere Technology (Suzhou) (Jiangsu, China). Both Cy5 mono‐reactive NHS Esters and DiI were purchased from Fanbo Biochemicals (Beijing, China).

Inactivated PRRS antigens were kindly provided by Luoyang Huizhong Animal Medicine. FMD antigenic VLPs were from Lanzhou Veterinary Research Institute. APC‐labeled anti‐mouse CD4 antibody, PE‐labeled anti‐mouse CD69 antibody, FITC‐labeled anti‐mouse CD8 antibody, Percp‐Cyanine5.5‐labeled anti‐mouse CD44 antibody and eFluor450‐labeled anti‐mouse CD62L antibody, ELISA kits for IL‐4 and IFN‐γ were purchased from eBioscience (California, USA). ELISPOT kits were obtained from Abcam (England). CCK8 kits were from Dojindo Laboratories (Japan). Other materials were all of analytical grade.

### 2.2 NPE preparation

**FIGURE 1:**
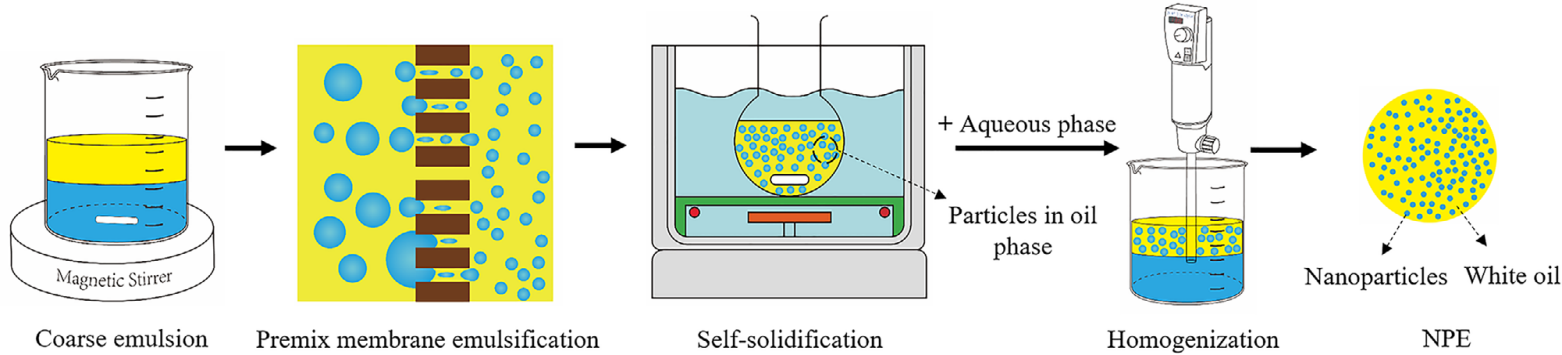
The schematic diagram of nanoparticles‐in‐emulsion (NPE) preparation. Coarse emulsion contained quaternized chitosan‐based solution and white oil‐based oil phase. After premix membrane emulsification and the self‐solidification, coarse emulsion was transformed into particles in oil phase. Then, NPE adjuvant was prepared by adding the outer aqueous phase and homogenizing the mixture

NPE were fabricated as shown in Fig. 1. Firstly, we prepared the oil phase containing quaternized chitosan particles as follows. Briefly, 0.3 g quaternized chitosan was dissolved in 1 M acetic acid, then GP aqueous solution was added into quaternized chitosan solution at 4°C. The quaternized chitosan‐based solution was further mixed with the white oil phase containing 1% PO500 under stirring, to obtain the coarse emulsion. The coarse emulsion was then pressed through porous membrane (1.2 µm) with proper pressure. Afterwards, the emulsion self‐solidified for 2 h at reasonable temperature and particles in oil phase were formed. Subsequently, oil dispersion of particles was dropped into the aqueous phase containing 2% Tween80 at a ratio of 2:1. The mixture was then emulsified by homogenization to obtain NPE.

### 2.3 Characterization of NPE

The size and size distribution of quaternized chitosan nanoparticles in oil phase were measured by a Zetasizer Nano dynamic light scattering analyzer (Malvern, UK). The uniformity of particles was characterized by polydispersity index (PDI). Good dispersibility of the particles was indicated if PDI < 0.3, while a satisfactory dispersibility was indicated if PDI < 0.1, which was reported before [14]. Tests were repeated at least three times.

In order to characterize the surface morphology of quaternized chitosan particles, the oil phase was removed by washing. The particle samples after drying were coated with platinum (10 mA × 120 s) under vacuum by a JFC‐1600 ion sputter (JEOL, Japan) and then observed by a JSM‐6700 scanning electron microscopy (SEM, JEOL, Japan).

The micro‐emulsion size was determined by Mastersizer2000 laser diffraction instrument (Malvern, UK). The volume diameters at 50% represented the average size of NPE. At least three parallel tests were analyzed. Span value lower than 1.0 indicated a narrow size distribution.

We characterized the shape and internal structure of NPE with the Olympus BX51 optical microscope (Olympus, Japan) and SP5 confocal laser scanning microscope (CLSM, Leica, Germany). To observe the intact structure under CLSM, chitosan and white oil was labeled by Cy5 and DiI, respectively.

**PRACTICAL APPLICATION**

In this article, we developed a novel formulation composed of hydrogel particles‐in‐emulsions by utilizing the self‐solidifying property of the temperature‐sensitive hydrogel system. Additionally, the organic oil phase used for this process was white oil, a common component in emulsion adjuvant, which avoided the washing steps and organic solvent residues. The entire process is green and easily scalable. Furthermore, the formulation design strategy of embedding particles in the oil phase significantly enhanced both humoral and cellular immunity compared with the other control groups. The new product has great potential for use in vaccine adjuvants. Moreover, the preparation method can be applied for preparing other combined bioformulation containing both particles and emulsions.

### 2.4 Vaccination study

Female Balb/c mice (*n* = 4, 6–8 weeks old) were purchased from Vital River laboratories (Beijing, China). Studies were performed in strict accordance with the Regulations for the Care and Use of Laboratory Animals and Guideline for Ethical Review of Animal (China, GB/T 35892‐2018). All animal experiments were reviewed and approved by the Animal Ethics Committee of the Institute of Process Engineering (approval ID: SYXK2019‐0004).

Mice were immunized from day 0 by intramuscular injection twice at a two‐week internal. Each group was injected 100 µL various formulations along with same amounts of antigens (80 µg PRRS antigens or 20 µg FMD VLPs). NPE vaccine was formed by simply mixing NPE adjuvant with prescribed antigens. It contained 50 µL mixture of oil phase and quaternized chitosan particles. The concentration of quaternized chitosan in this mixture was 1.2 µg/µL. Nanoparticles (NP) vaccine was prepared by mixing the same quaternized chitosan nanoparticles with antigens. The nanoparticles were separated from particle‐embedded oil phase as reported [15]. ISA206 formulation, in which the volume of oil phase was 50 µL, was prepared according to the instruction guide. Serum was collected on day 14 and day 28. After centrifugation, sera were separated from serum and stored at ‐80°C. On day 35, mice were sacrificed and the spleen were harvested for further analysis.

### 2.5 Evaluation of immune responses

#### 2.5.1 Antibody determination

Antigen‐specific sera antibodies were determined by ELISA according to the protocol described previously [16, 17]. Briefly, ELISA plates were coated with 100 µL per well of PRRS antigens (20 µg/mL) or FMD VLPs (2 µg/mL) in coating buffer and incubated at 4°C overnight. Then the plates were blocked with 1% w/v BSA in PBS for more than 1 hour at 37°C after washed three times. Subsequently, appropriate sera dilutions were added into the washed plates with an initial 100‐fold dilution and serial two‐fold dilutions. Plates were incubated at 37°C for 1 hour and washed 4 times. Thereafter, the plates were stained with 100 µL HRP‐conjugated IgG antibodies (1:10000), IgG1 antibodies (1:50000) or IgG2a antibodies (1:50000) and incubated at 37°C for 45 min. Tetramethylbenzidine (TMB) substrate was added into the washed plates and the chromogenic reaction was stopped by adding H_2_SO_4_. The OD450 values were detected by using a Tecan microplate reader. The antibody titers were regarded as the sample dilution with an OD450 equal to twice the mean negative sera well.

#### 2.5.2 Splenocytes proliferation

Splenocyte proliferation was assayed according to previously described method [18]. In brief, splenocytes were cultured in triplicate in 96‐well plates in the absence or presence of FMD antigens (5 µg/mL) for 36 hours. CCK8 solution was added to each well (10 µL/100 µL culture medium). Then another 3 hours’ incubating for the plate followed while keeping it away from light. The absorbance was obtained at 450 nm (A_450_), and at 600 nm as reference wavelength. The stimulation index (SI) was calculated as SI = (A _stimulated_‐A_blank_)/ (A _non‐stimulated_‐A_blank_).

#### 2.5.3 Cytokines secreting lymphocytes

Frequencies of IFN‐γ secreting cells were measured as described before [19]. The 96‐well PVDF‐based membrane plates were coated with 15 µg/mL monoclonal antibody overnight at 4°C. 200 µL culture medium was added in each well for blocking. Then splenocytes were incubated with 25 µg/mL PRRS antigen in medium for 18 hours. After incubation, the splenocytes were removed and the plates were incubated with 200 µL/well cold water for 10 min at 4°C. Later, the washed plates were incubated with a biotinylated antibody and Streptavidin‐Alkaline phosphatase, successively. Then distinct spots emerged after 100 µL/well substrate solution was added for 10 min. The plates were then washed extensively with water and the spots were counted using an ELISPOT reader (AT‐Spot 3300, China).

#### 2.5.4 Cytokines secretion

After spleens were harvested, splenocytes were collected by grinding through a 70 µm cell strainer and removing the erythrocytes. We cultured the splenocytes at the concentration of 2 × 10^6^ cells per mL with FMD antigens (5 µg/mL) for 60 h. After centrifugation, the supernatants were obtained and stored at ‐80°C until analyzed. The concentration of cytokines including IFN‐γ and IL‐4 were measured by using ELISA kits in accordance with the manufacturer's protocol.

#### 2.5.5 Lymphocytes evaluation

Splenocytes were collected from PRRS antigen‐immunized mice. Flow cytometry assay was conducted to examine the lymphocytes activation and memory T cell response in splenocytes. After stimulated with antigens (25 µg/mL) for 60 hours, splenocytes were collected and stained with fluorochrome‐conjugated anti‐mouse antibodies for 30 min at 4°C. After washed, cells suspensions were examined using a flow cytometer (BD LSRFortessa, USA).

### 2.6 Biosafety assessment

The in vitro cytotoxicity assay was carried out with mouse bone marrow‐derived dendritic cells (BMDCs) [20]. The erythrocytes were removed and remaining cells were incubated for 6 days in the culture medium with 10% fetal bovine serum, 10 ng/mL GM‐CSF, 20 ng/mL IL‐4, 100 µg/mL streptomycin and 100 unit/mL penicillin. The medium of cultures was changed every other day by half. On day 6, BMDCs were collected and counted with a scepter cell counter (Merck Millipore, USA). 100 µL BMDCs were seeded in each well of 96‐well plates at a concentration of 10^6^ cells/mL. After overnight culture, BMDCs were incubated with various volumes of emulsions for 24 h. Then, the supernatant was removed. Subsequently, a mixture of CCK8 solution and culture medium at a proportion of 1:10 was added to each well and incubated for another 3 hours. The absorbance at 450 nm was detected using the microplate reader. And the results of treatments were expressed as a percentage of the control.

Balb/c mice were injected intramuscularly with ISA206 or NPE to evaluate the local inflammation *in vivo*. Every day two mice in each group were killed. The residual emulsion and lesions in the local muscle were observed after dissection.

### 2.7 Statistical analysis

All statistical analysis was performed using GraphPad Prism 6 software. And results were expressed as means ± standard error. The *p* values between two groups were obtained using an unpaired, two‐sided Student's t test. Differences among more than two groups were evaluated by one‐way ANOVA and Tukey's multiple comparison. Statistically significant was expressed as follows: *p < 0.05, **p  < 0.01, ***p  < 0.001, ****p  < 0.0001.

## 3 RESULTS AND DISCUSSION

### 3.1 Optimization and characterization of particles

To obtain the particles‐in‐oil‐in‐water formulation, we first optimized the properties of particles. The particles were prepared using a thermo‐sensitive quaternized chitosan‐GP formulation, which is in a liquid state at room temperature and transforms into a gel state at temperatures over 37°C. We utilized this phase transition to prepare the particles without using additional cross‐linking agents. We have successfully obtained particles by this method in our previous work using the mixture of liquid paraffin/petroleum ether as oil phase [12]. In this study, white oil was used to replace the mixture of liquid paraffin/petroleum ether as the continuous phase and therefore the washing step can be eliminated.

Because the use of a different oil phase affected the emulsification process and the size of the resulting particles, we optimized the main processing parameters, such as the oil‐water ratio, the stirring speed, and solidification temperature. Among them, oil‐water ratio and stirring speed, both of which directly affect the phase parameters, are highly relevant in the premix membrane emulsification [21]. For example, uniform particles cannot be obtained with very high premix stirring speed even if the oil‐water ratio is on its optimal level [22]. Here, we devoted to obtain as small and uniform particles as possible, as requested by vaccine preparation below. Thus, we tried to prepare satisfactory particles when investigating a single factor. As shown in Table 1, when the oil‐water ratio was as low as 20:1, the droplets tended to aggregate during solidification and the resulting uniformity was poor. When the oil‐water ratio was 30:1, the viscosity of the oil phase was too low, and some droplets passed through the membrane pores easily and were not broken up in time. Particles with a satisfactory PDI were obtained when the oil‐water ratio was 25:1.

**TABLE 1:** Sizes and PDI of prepared particles with different oil‐water ratios

**Oil‐water ratio**	**Particle size (nm)**	**PDI**
20:1	728±16	0.176
25:1	531±11	0.079
30:1	771±33	0.396

**FIGURE 2:**
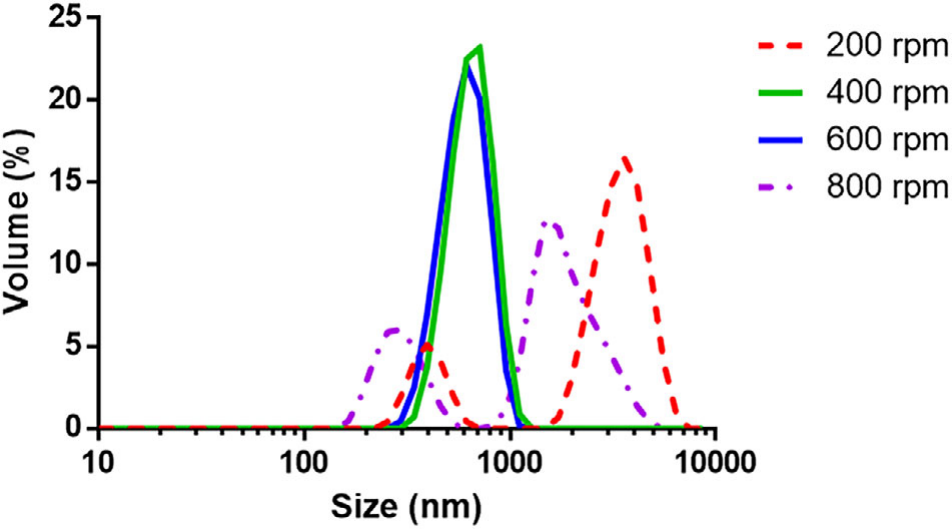
Size distributions of particles prepared by various stirring rates

The premix stirring speeds had significant effects on the sizes of the formed droplets (Fig. 2). When the stirring speed was as low as 200 rpm, the sizes of droplets in coarse emulsion were very large and it was not easy to break into a fine emulsion. However, raising the stirring speed to 800 rpm resulted in the coarse emulsion droplets that were smaller than the diameter of membrane pores, resulting in poor uniformity of the fine emulsion, which led to coalescence and a broad size distribution of the final particles. Therefore, the optimal stirring speed was between 400 rpm and 600 rpm.

**FIGURE 3:**
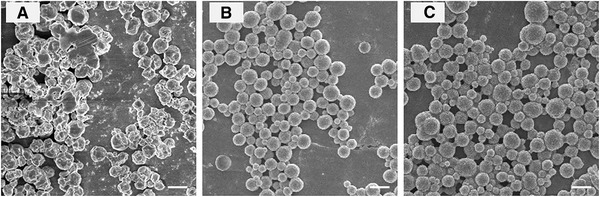
The SEM images of prepared particles at different solidification temperature. (A) 40°C. (B) 60°C. (C) 80°C. Scale bar = 1 µm

The solidification temperature significantly affected the morphology and the mechanical strength of the quaternized chitosan particles. When the temperature was as low as 40°C, the mechanical strength of the formed porous hydrogel was low, resulting in the collapse and aggregation of particles (Fig. 3A). Hydrogel particles with a compact structure were obtained under the solidification conditions of 60°C and 80°C as displayed in Fig. 3B and Fig. 3C, respectively. Considering the higher energy consumption and greater inconvenience in operating equipment at 80°C, 60°C was selected as the most suitable solidification temperature.

**FIGURE 4:**
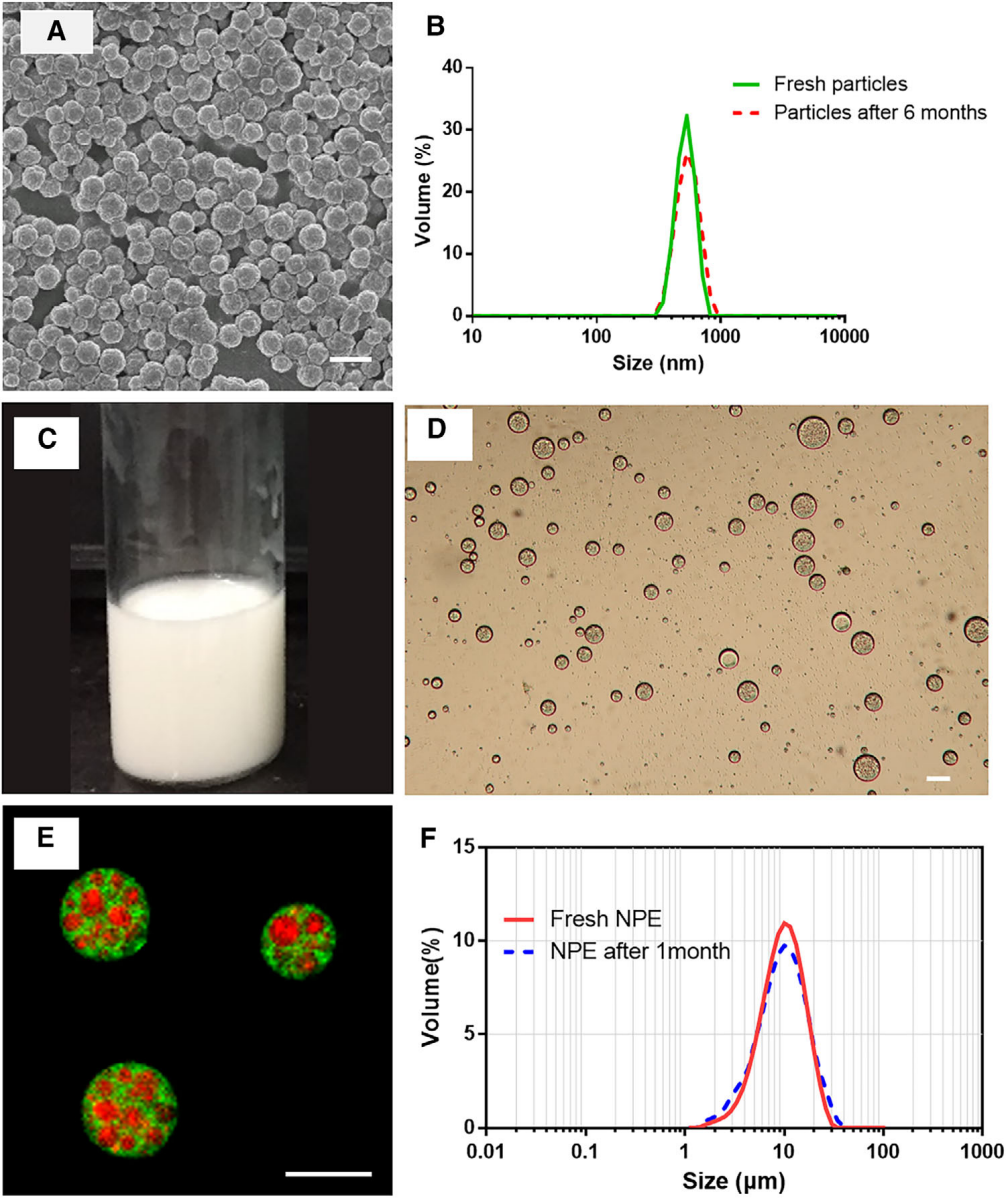
Characterizations of optimized particles and nanoparticles‐in‐emulsion (NPE). (A) SEM images of particles. Scale bar = 1 µm. (B) Size distributions of particles dispersed in oil phase before and after storing 6 months. (C) The visual image of NPE. (D) The optical image of NPE. Scale bar = 10 µm. (E) The confocal image of NPE. The white oil and chitosan were labelled with DiI (green)and Cy5 (red), respectively. Scale bar represents 10 µm. (F) Size distributions of NPE prepared freshly and stored after 1 month

After optimization, quaternized chitosan nanoparticles with excellent uniformity and an average size of approximately 500 nm were obtained (Fig. 4A). The nanoparticles could be stored directly in the oil phase. Furthermore, even after being kept at 25°C for 6 months, the particles still possessed a narrow size distribution (Fig. 4B).

### 3.2 Preparation and characterization of NPE

NPE were prepared by emulsifying the mixture of particle‐embedded oil phase and the aqueous phase. Here, we optimized the rate of homogenization, which had significant effects on the size of NPE. The preparation results of NPE with different homogenization rates are shown in Table 2. The higher speed resulted in NPE with smaller size. When the speed reached 30000 rpm, the amount of droplet breakage was too high, causing broad size distribution of NPE. Thus, the homogenization rate of 24000 rpm was chosen to prepare NPE with an average size of 9.107 ± 0.359 µm.

**TABLE 2:** Sizes and span values of NPE prepared by different homogenization speeds

**Homogenization speed (rpm)**	**Emulsion size (µm)**	**Span**
12 000	44.006±0.148	0.775
18 000	18.178±0.085	0.690
24 000	9.106±0.359	0.683
30 000	8.359±0.222	1.305

After optimization, a milky NPE emulsion was produced successfully (Fig. 4C). Optical microscopy and laser confocal microscopy revealed that the prepared emulsion NPE had an oil‐in‐water structure (Fig. 4D), and that the nanoparticles were loaded inside the droplets (Fig. 4E). As shown in Fig. 4F, the NPE remained stable after storage for 1 month at 4°C.

### 3.3 Evaluation NPE's adjuvanticity in PRRS and FMD vaccines

To evaluate the immune‐potentiating effects of NPE in veterinary vaccines, we chose two representative antigens. One was the inactivated antigen against PRRS virus, and the other antigen selected was the FMD VLPs. Both PRRS and FMD are the highly contagious diseases of great economic significance in the livestock industry worldwide, which highlights the importance of targeted vaccine development [23, 24]. Their most commercial vaccines contain oil‐based adjuvants [25, 26]. And inactivated antigens are the most commonly used in vaccines, while VLPs represent the promising vaccine candidates [27, 28]. But their immunogenicity is less satisfactory currently. Therefore, we investigated the adjuvant activity of NPE in both vaccines for evaluating its efficacy and improving its adaptability.

**FIGURE 5:**
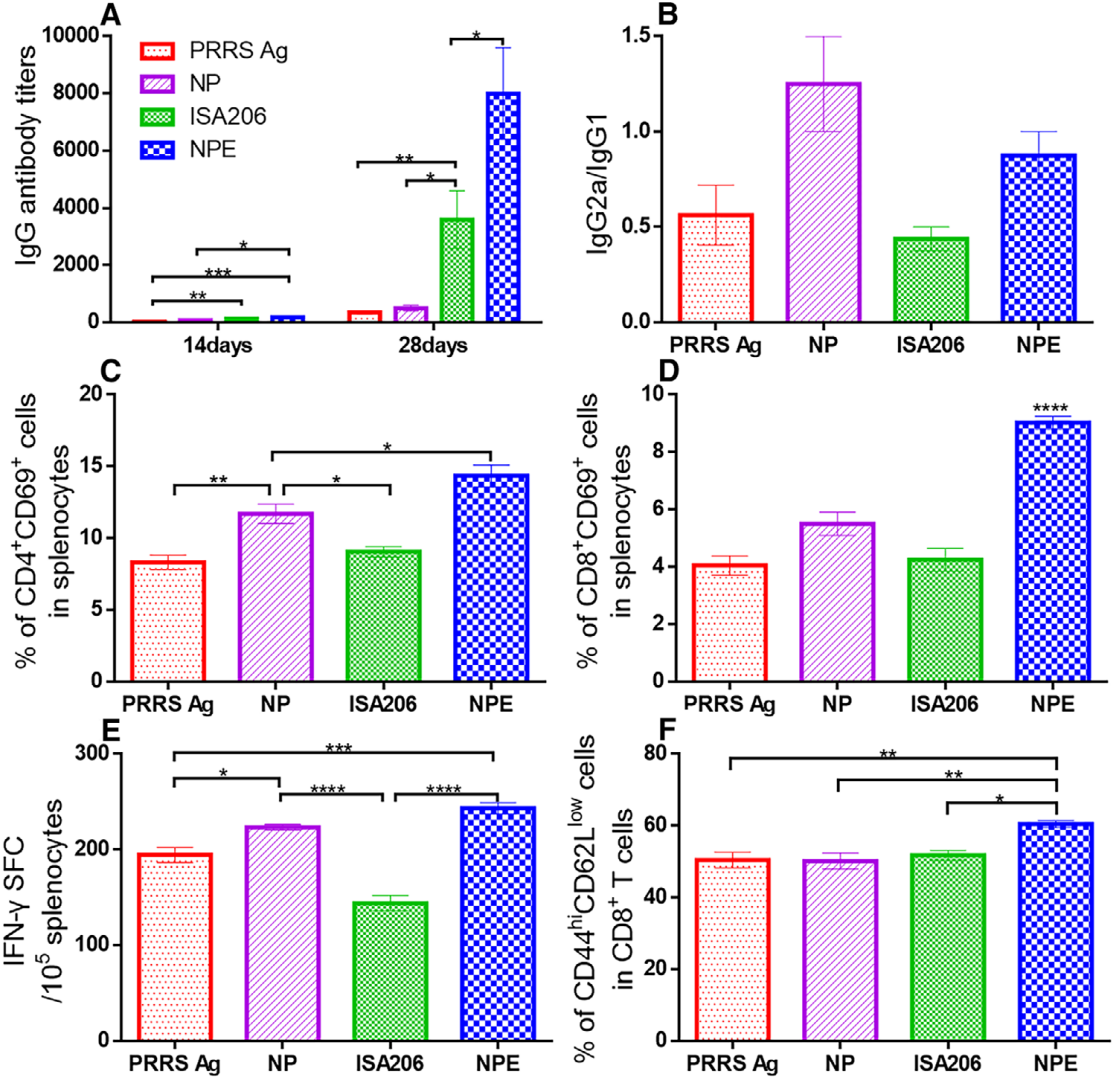
Vaccination efficacy against PRRS enhanced by nanoparticles‐in‐emulsion (NPE). Mice were intramuscularly injected with PRRS antigen alone (PRRS Ag), with nanoparticles (NP), with ISA206 (a kind of commercial adjuvant), or with NPE on day 0, followed by a boost with the same doses on day 14. Serum was collected on day 14 or on day 28 and analyzed for IgG, IgG2a and IgG1 antibodies. The mice were sacrificed on day 35 and spleens were harvested for flow cytometry and ELISPOT analysis. (A) PRRS‐specific IgG antibody titers. (B) The ratio of PRRS‐specific IgG2a and IgG1 levels on day 28. (C) Frequencies of CD4^+^CD69^+^ T cells in the splenocytes. (D) Frequencies of CD8^+^CD69^+^ T cells in the splenocytes on day 35. (E) ELISPOT analysis of IFN‐γ secreting cells among splenocytes. SFC represents the spot forming cell. (F) Flow cytometry determination on the expression of CD44^hi^CD62L^low^ in CD8^+^ T cells. *p < 0.05, **p < 0.01, ***p < 0.001, ****p < 0.0001

IgG antibody titers in sera collected on days 14 and 28 were measured to evaluate the humoral immunity (Fig. 5A and Fig. S2A). The antibody levels here were significantly increased only in oil emulsion vaccines including ISA206 and NPE from day 14 to day 28. And NPE vaccination generated the highest IgG antibody titers. For PRRS vaccination, NPE significantly increased antibody titers in comparison with either antigen alone or ISA206 (Fig. 5A). And NP induced limited increase in the antibody titers compared to PRRS antigen alone. For FMD vaccination, NPE showed a significantly different response from that elicited by FMD antigen alone, and insignificant response compared to ISA206 (Fig. S2A). Altogether, the results indicated that NPE could be almost equally, or potentially more effective than ISA206 in enhancing humoral immunity. In addition, NPE switched the immune response from Th2‐bias towards expected Th1‐direction, indicated by a higher IgG2a/IgG1 ratio than that of ISA206 [29], reflecting potent cell‐mediated (Th1) immune response (Fig. 5B and Fig. S2B). And the highest IgG2a/IgG1 ratio in the NP group provided evidence that the addition of chitosan nanoparticles in NPE successfully modulated the Th1/Th2 balance.

To further investigate the influence of NPE on cell‐mediated immune responses, splenocytes were cultured and analyzed from following aspects: lymphocyte proliferation/activation (the essential premise), cytokines secretion (the magnitude) and memory lymphocytes generation (the duration) [30, 31]. Among them, for PRRS vaccination, although no significant difference in lymphocyte proliferation was observed (Fig. S1A), the group vaccinated with NPE presented significantly increased activation of both CD4^+^T cells and CD8^+^T cells, marked by the expression of CD69 [32], compared with the other groups (Fig. 5C and Fig. 5D). For FMD vaccination, significant lymphocyte proliferation and insignificant activation were noted (Fig. S2C‐E), explained by the truth that VLPs antigen alone are capable of activating T cells due to their mimicry of the epitopes present on the native virus [33]. Therefore, these results suggested that NPE could enhance the cell immune response by activating or proliferating lymphocytes.

Under the premise of effective lymphocyte proliferation or activation, the magnitude of cell‐mediated responses was measured, reflected by the key cytokines IFN‐γ (Th1 cytokine) and IL‐4 (Th2 cytokine). The ELISPOT assay showed that most IFN‐γ‐secreting cells were observed after NPE immunization, followed by NP immunization (Fig. 5E), as evidence by the fact that chitosan promoted cell‐mediated immunity via inducing rich IFN‐γ and emulsion could potentiate the IFN‐γ induction along with Th1‐biased adjuvants [34, 35]. Combining with the significant increase of IL4‐secreting cells in both NPE and ISA206 group (Fig. S1B), these results indicated that NPE could efficiently enhance both Th1 and Th2 immune responses. Similar results were observed by evaluating the secreted concentrations of IFN‐γ and IL‐4 for FMD vaccination (Fig. S2F‐G), confirmed that conclusion once again.

Moreover, the duration of cell‐mediated responses here determined by the production of effector memory T cells, which can rapidly proliferate and differentiate into effector cells that respond quickly and effectively to viral infections [36]. The proportion of effector memory T cells, expressing high level of CD44 molecules and low level of CD62L molecules, was detected by flow cytometry. Evidently a much higher proportion of effector memory CD8^+^ cells (Fig. 5F) than all other vaccines and an insignificant percentage of effector memory CD4^+^ cells (Fig. S1C) compared with the NP group, were generated after NPE immunization. Similar to the results observed for the PRRS vaccines, NPE induced the highest increase in the production of effector memory CD8^+^ cells and no reduce in the frequency of effector memory CD4^+^ cells (Fig. S2H‐I). These results demonstrated the potential of NPE for long‐term cell‐mediated immune response. In combination, the superior cellular immunity elicited by NPE was validated.

Collectively, these findings showed that NPE provoked robust humoral and cell‐mediated immune responses against PRRS or FMD. Meanwhile, NP and ISA206 induced weaker humoral immunity and cellular immunity, respectively. Admittedly, the primary reason for NPE vaccine efficacy is that the addition of chitosan nanoparticles into emulsion modulated the immune response and resulted in a synergistic effect. However, it could not be ignored that NPE, with nanoparticles embedded in the emulsion, induced immunity beyond expectation, distinct from that of simply mixed formulation reported before [9]. It has been reported that emulsions resulted in local immunocompetent environment at the injection site, while nanoparticles mainly led to cellular uptake and intracellular trafficking [37, 38]. Therefore, weaker immunity induced by mixed formulation was evidenced by the different action mechanisms and temporal distributions of emulsions and nanoparticles on their own alone. As a result, NPE's special formulation, with particles and emulsions as a whole, presumably, led to a total different distribution and mechanism in vivo from ISA206, which further enhanced the immune response in vaccines.

### 3.4 Preliminary biosafety studies of NPE *in vitro* and *in vivo*

**FIGURE 6:**
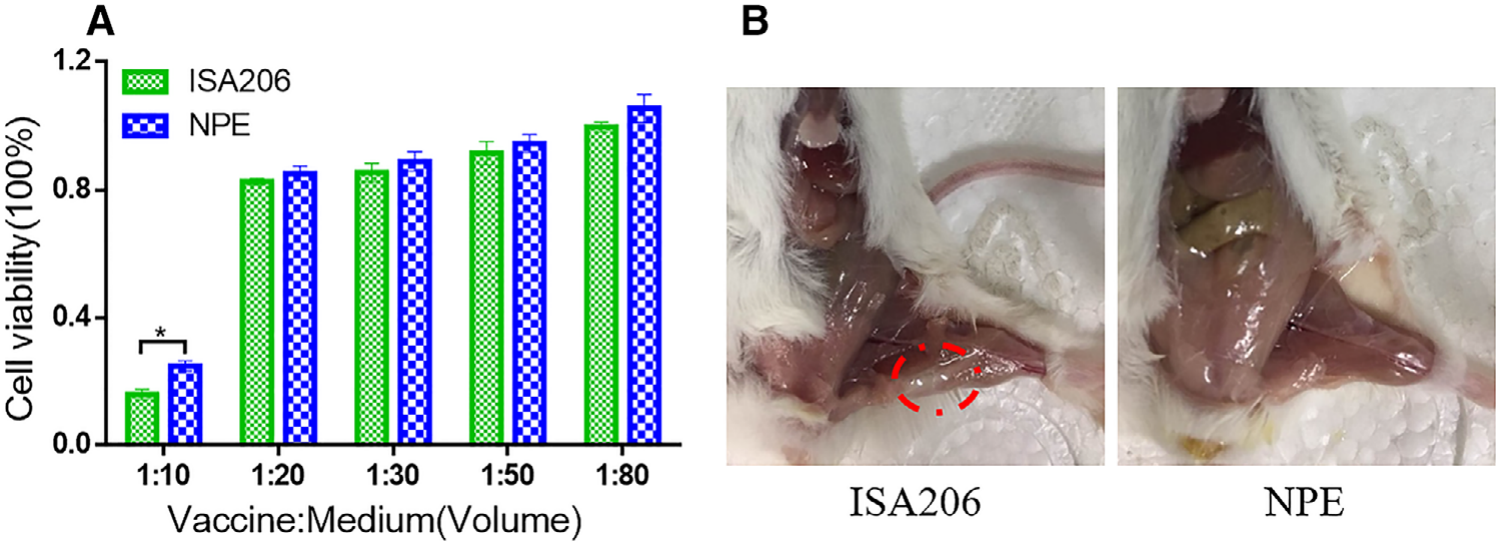
Biocompatibility of nanoparticles‐in‐emulsion (NPE) in vitro and in vivo. (A) The bone marrow‐derived dendritic cells (BMDCs) were incubated for 24 hours with various volumes of the indicated formulations (NPE or commercial adjuvant ISA206). The BMDCs viability was detected by in vitro cytotoxicity assay. (B) Mice were injected in the inner thigh with ISA206 or NPE. And representative images of local inflammation induced by each adjuvant on day 7 in vivo were shown

Biosafety assessment is as important as efficacy evaluation for veterinary adjuvants. Poor vaccine safety affects not only animal growth, but also food quality. Thus, we assessed the biosafety of NPE by evaluating cytotoxicity (Fig. 6A) and local histological changes (Fig. 6B). As shown in Fig. 6A, BMDCs viability decreased gradually as the concentration of vaccine in the culture medium increased. When the concentrations of both emulsions were lower than 5% (v/v), the BMDCs could maintain their viability over 80%. Even the volume of both emulsions added into the cell supernatants were increased to a high level, the survival rate in the NPE group was still higher than that in the ISA206 group. This could be explained by the fact that surfactants, the main components causing cytotoxicity, were at a high concentration for self‐emulsifying purpose in the ISA206 formulation [39].

Moreover, as shown in Fig. 6B, the local tissue of group ISA206, which is marked by a red circle, formed vesicular exanthema, while this was not visible in the NPE group. In fact, NPE had disappeared from the injection site by day 7. Injection site reactions are of great concern in food‐producing animals because they lead to a decrease in the quality of the meat. Research has shown that the W/O/W emulsion leaves great amounts of residues at the injection site [40]. In contrast, O/W emulsions have been shown to induce little local reaction owing to their fluidity and good tolerance [41]. Therefore, the NPE vaccine showed a great potential as an alternative adjuvant.

## 4 CONCLUDING REMARKS

Emulsions, the most commonly used type of adjuvant in veterinary vaccines, induce poor cellular immunity, furthermore, the washing operation and solvent residue in the conventional particle preparation processes limited the further scale‐up production of particles. And simply adding particles to the aqueous phase of emulsion doesn't produce synergistic immune‐enhancing effects. Therefore, we developed a green and simple process for fabricating a nanoparticles‐in‐emulsions formulation. The particles were prepared by introducing a self‐solidifying hydrogel system, without adding additional crosslinking reagents. In addition, the preparation only used white oil as the oil phase without adding other solvents, which successfully prevented additional cumbersome particle washing steps and organic solvent residues. Finally, an optimal quaternized chitosan particles‐in‐oil‐in‐water emulsion adjuvant was produced. Subsequently, we evaluated the effectiveness of this adjuvant using two typical antigens against two animal diseases. The adjuvant was mixed with antigens before use. Highly effective humoral and cellular immune responses were achieved for the two vaccines, PRRS inactivated antigen and FMD VLPs. The particles‐in‐emulsions has great potential in improving vaccine preparation and producing an enhanced immune effect.

This type of particles‐in‐oil‐in‐water emulsion not only induced humoral immunity better than emulsion adjuvants, but also elicited excellent cellular immunity similar to particle vaccines. This combination can be extended to other veterinary adjuvants or human vaccine adjuvants. Furthermore, the platform for preparing a bioformulation containing both particles and emulsions can be used for other vaccine applications or drug delivery systems.

## CONFLICT OF INTEREST

The authors have declared no conflict of interest.

## Supporting information

Supporting Information
