# Supplementary material for: Green preparation of hydrogel particles‐in‐emulsions for simultaneous enhancement of humoral and cell‐mediated immunity
Source: Eng Life Sci. 2020 Sep 21;20(11):514–24. doi: 10.1002/elsc.202000011 (PMC7645649; doi:10.1002/elsc.202000011)
Supplement: Supplementary file 1 — Supporting Information [file ELSC-20-514-s001.pdf]

Supplementary Information

for

Green preparation of hydrogel particles-in-emulsions for simultaneous enhancement of humoral and cell-mediated immunity

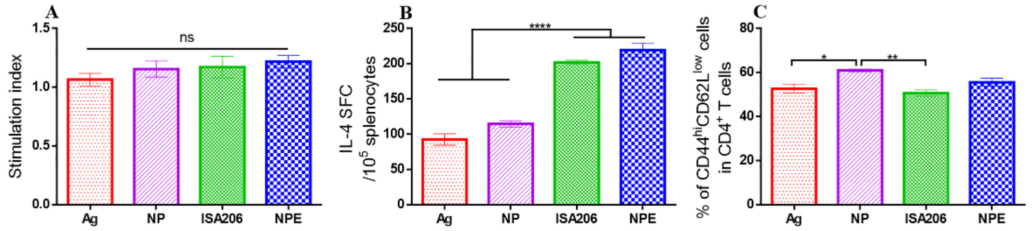

Fig S1. Vaccination efficacy against PRRS enhanced by nanoparticles-in-emulsion (NPE). Mice were intramuscularly injected with PRRS antigen alone (PRRS Ag), with nanoparticles (NP), with ISA206 (a kind of commercial adjuvant), or with NPE at a two-week internal. The mice were sacrificed on day 35 and spleens were harvested for analysis. (**A**) The proliferation levels of spleen cells. (**B**) ELISPOT analysis of IL-4 secreting cells among splenocytes. SFC represents the spot forming cell. (**C**) Flow cytometry results on $CD4^+$ T cells for the expression of $CD44^{hi}CD62L^{low}$. *P<0.05; **P<0.01; ****P<0.0001; ns, statistical non-significance.

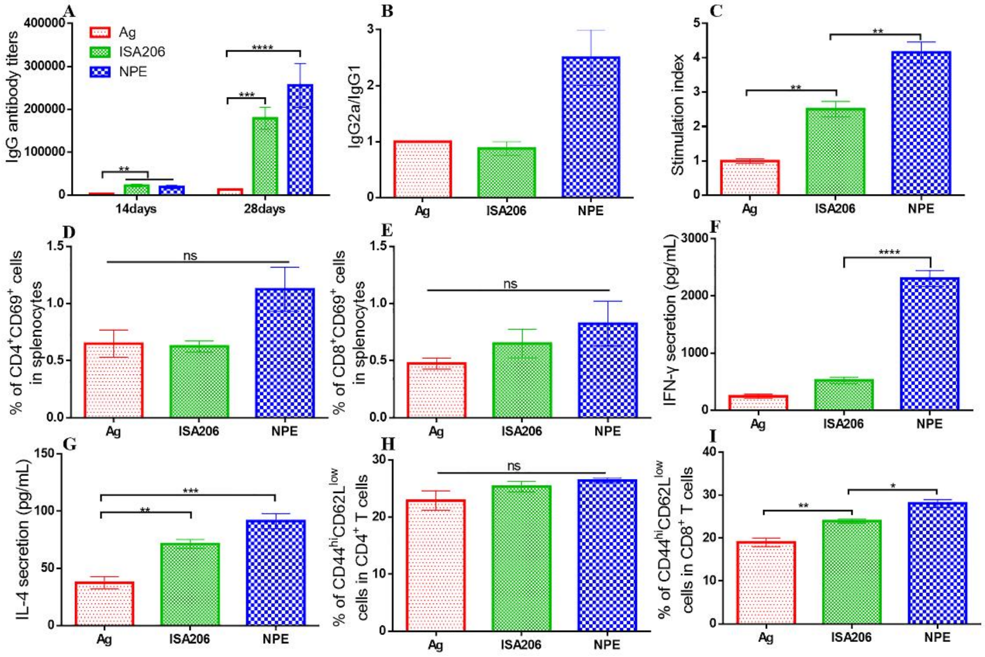

Fig S2. Vaccination efficacy against FMD enhanced by nanoparticles-in-emulsion (NPE). Mice were intramuscularly injected with FMD antigen alone (FMD Ag), with nanoparticles (NP), with ISA206 (a kind of commercial adjuvant), or with NPE on day 0, followed by a boost with the same doses on day 14. The mice were sacrificed on day 35 and spleens were harvested for further analysis. (**A**) Serum FMD antigen-specific IgG antibodies on day 14 and day 28. (**B**) The ratio of FMD antigen-specific IgG2a and IgG1 levels on day 28. (**C**) Spleen cells proliferation measured by the CCK8 method. (**D**) Activated $CD4^+$ T cells indicated by $CD69^+$ molecules. (**E**) Flow cytometry analysis on the expression of $CD69^+$ in $CD8^+$ T cells. (**F**) IFN-γ release in the culture supernatant of spleenocytes. (**G**) IL-4 secretion in the cell culture supernatant. (**H**) Proportions of $CD44^{hi}CD62L^{low}$ in $CD4^+$ T cells. (**I**) Flow cytometry evaluation of $CD44^{hi}CD62L^{low}$ in $CD8^+$ T cells. $^*P<0.05$; $^{**}P<0.01$; $^{****}P<0.0001$; ns, statistical non-significance.
